# Perception of the primary health care response capacity by patients with and without mental health problems, and health professionals: qualitative study

**DOI:** 10.1186/s12913-021-06205-w

**Published:** 2021-03-31

**Authors:** Eva Rodríguez-Eguizabal, Bárbara Oliván-Blázquez, Valle Coronado-Vázquez, Mª. Antonia Sánchez-Calavera, Mª. Josefa Gil-de-Goméz, Sergio Lafita-Mainz, África Garcia-Roy, Rosa Magallón-Botaya

**Affiliations:** 1 Health Service of La Rioja, Primary Health Center Arnedo, Av de Benidorm, 57, Arnedo, La Rioja 26580 Spain; 2 Health Research Institute of Aragon (IIS Aragón), Edificio CIBA, Avda. San Juan Bosco, 13, Zaragoza, 50009 Spain; 3 Research network on preventive activities and health promotion (Red de Investigación en Actividades Preventivas y Promoción de la Salud) (RedIAPP), Gran Via de les Corts Catalanes, 587, Barcelona, 08007 Spain; 4 Department of Psychology and Sociology, University of Zaragoza, Violante de Hungría 23, Zaragoza, 50009 Spain; 5 Aragonés Health Science Institute, Avda. San Juan Bosco, 13, Zaragoza, 50009 Spain; 6 Health Service of Castilla La Mancha. Primary Health Center Illescas, C/ Sandro Pertini S/N. 45.200, Toledo, Illescas Spain; 7 Aragones Health Service, Plaza de la Convivencia, 2, Zaragoza, 50017 Spain; 8 Department of Medicine and Psychiatry. University of Zaragoza, Domingo Miral, S/N, Zaragoza, 50002 Spain; 9 Health Services of La Rioja, Teaching Unit of San Pedro Hospital, San Pedro. C/ Piqueras 98, 26006 Logroño, Spain

**Keywords:** Responsiveness, Primary Health care, Mental health

## Abstract

**Background:**

The objective of this study is to deepen our understanding of perceptions towards Primary Health Care Response Capacity by specifically using patients with and without mental disorders, as well as family doctors and a manager, in order to compare and endorse perspectives. For it, a qualitative study was performed. In-depth interviews were conducted with 28 patients with and without mental health disorders and focus groups were held with 21 professionals and a manager. An inductive thematic content analysis was performed in order to explore, develop and define the emergent categories of analysis.

**Results:**

The fundamental domains for patients are dignity, communication, and rapid service. People with mental health problems also highlight the domain of confidentiality as relevant, while patients who do not have a mental health problem prioritize the domain of autonomy. Patients with mental health disorders report a greater number of negative experiences in relation to the domain of dignity. Patients do not consider their negative experiences to be a structural problem of the system. These findings are also endorsed by health care professionals.

**Conclusions:**

It is necessary to take these results into account as responsive systems can improve service uptake, ensure adherence to treatment, and ultimately enhance patient welfare.

## Background

Globally, mental health diseases are a major problem. In fact, according to the WHO, 450 million people suffer from a mental or behavioral disorder, with every 1 in 4 people suffering from a mental disorder (MD) at least once in their life, and with mood disorders and depression being the most frequent [1–5]. From a health care perspective, Primary Health Care (PHC) is considered the most effective in the management and treatment of these mental disorders [1]. Several studies have confirmed that 25–35% of patients receiving PHC services have a psychiatric condition, with over 80% of these patients experiencing depression or anxiety disorders. In fact, 53.6% of the patients in primary health care settings present one or more psychiatric disorders, with the most prevalent being affective (35.8%), anxiety (25.6%), and somatoform (28.8%) disorders. There were 30.3% of patients who had more than one mental disorder at the time of the study and 11.5% presented comorbidity between affective, anxiety, and somatoform disorders [6]. Furthermore, it is widely known that General Practitioners (GPs) only refer approximately 5–10% of their psychiatric patients, whose disorders were detected in Primary Care, to Mental Health Services [7]. This is due to PHC being considered the main health platform where mental health patients are treated.

Since the declaration of Alma-Ata, Primary Care has been considered a fundamental part of the health system, as it is seen as the most accessible level of care to the individual and community. It is based on effective methods and technologies and is provided at an affordable cost to society [8]. Furthermore, it is the level of care closest to the health determinants of the individuals and the community it serves [9]. This allows for it to address the multi-causality of diseases and maintain a bio-psycho-social approach, intersectorality, and comprehensive care. However, changes in society have also brought out changes in the position of the general population regarding their care and decision-making about their own health [10].

Another aspect to take into account is the quality of the healthcare given, with reference to both clinical and non-clinical care [11]. Regarding the latter, the WHO’s report ‘The world health report 2000 - Health systems: improving performance’ [12], measures how well a health care system meets the legitimate expectations of the population with regards to the non-health-enhancing aspects of the health system. The report was the first to develop the concept of ‘Health Systems’ Response Capacity (HSRC), which is defined as the “ability of the health system to meet the population’s legitimate expectations regarding their interaction with the health system, apart from expectations for improvements in health or wealth”. It may be defined as the way in which people are attended to and the environment in which they are treated. It values the personal experience of the patients when in contact with the health care environment [13] and is different from patient satisfaction with the care they receive. HSRC is measured through eight domains which are in accordance with the framework of human rights, and which are classified into two main categories: the domains that relate to respect for a person (which include dignity, confidentiality, communication, and the autonomy of individuals; and the structure-oriented domains (which include rapid service, basic quality of facilities, access to social support networks, and choice of care provider) [14]. These domains, shown in Table 1, were established for both the hospital setting and primary care services. However, the domain of social support refers only to the interaction with hospital care, so would not apply to primary health care. Understanding the opinions and perceptions of health care service users regarding the quality of care is essential for promoting changes and implementing effective measures for improvement.

**Table 1:** Definition of the domains of the response capacity of the WHO

DOMAIN	DEFINITION
Dignity	To be treated respectfully by the personnel of the health service. Maintain privacy during physical examinations and treatment
Confidentiality	To carry out visits in a manner that ensures privacy. Respect the confidential nature of the information about the illness.
Autonomy	Involve the patient, if they so wish, in the decisions regarding care and treatment and allow the patient to reject these, unless suffering from a deterioration of their mental faculties. Seek permission before applying any tests or treatments.
Rapid Service	Ensure rapid service in cases of emergency. Short waiting times for visits, tests, treatments and hospital admission. Availability of health personnel when hospitalization is required.
Clear Communication	Provide patients with information about their problem in understandable terms. Maintain close dialog between patients and providers. Listen attentively. Allow patients and their families enough time to ask questions.
Choice	Free choice of providers and services.
Social support	Family and friends can take hospital patients their favorite meals and soaps. Allow interaction with family and friends. Allow patients to observe religious practices.
Quality of Basic Services	Availability of generous spaces. Clean environment. Appropriate furniture, sufficient ventilation, clean restrooms.

Source: Own

Given the percentage of patients who suffer from a mental health disorder, and who are attended to mainly by primary health care services, it is relevant to analyze the patient perception of the Primary Health Care Response Capacity (PHCRC). This is due to an efficient response capacity being able to contribute to improving health, fostering promotion and prevention, and facilitating interaction and communication with healthcare providers in a more efficient way [15]. Furthermore, as stated by WHO in its ‘Mental Health Action Plan 2013-2020’ [16], health systems have not yet adequately responded to the burden of mental disorders and, therefore, the gap between the need for treatment and the provisions provided is considerable all over the world.

In addition to being an intrinsic goal, responsiveness is also a process involving multiple interactions within health systems and has an integral value within health systems. Improved responsiveness, while itself being a health systems goal, also contributes towards equitable improvements in population health. Responsive systems can improve service uptake, ensure adherence to treatment, and ultimately enhance patient welfare [17]. People, especially the most vulnerable (and people with mental disorders are among this vulnerable population), are more likely to use services if health systems are responsive to their expectations. Conversely, people are unlikely to use services within unresponsive systems [18]. Yet, of the four intrinsic goals of health systems, that is, improving health, ensuring fairness in financing, efficiency, and responsiveness, it is responsiveness that is the least studied [17]. Besides, to our knowledge, there are few studies that analyze the opinions of patients with mental health disorders towards the HSRC [19–22], and furthermore, those that do, do not focus specifically on PHC.

## Methods

The objective of this study is to deepen our understanding of opinions towards PHCRC by specifically using patients with and without mental disorders, as well as family doctors and a manager, with the goal of assessing the most effective domains for patients in terms of responsiveness and assessing their satisfaction in each domain. Another objective of this study is to analyze the similarities and differences between the perspectives of physicians regarding their patients with mental health problems and the perspectives of patients with and without mental health problems. The study also intends to analyze whether there is a difference in the perception of PHCRC according to the gender of the interviewees.

A qualitative design was carried out in order to collect information from an intentional sample of PHC patients with and without mental disorders, GPs, and from a member of a PHC management team. In-depth interviews and discussion groups were used to collect subjective data and to access understanding for the processes involved in generating expectations [23]. The individual interviews and the discussion groups were held by two interviewers, both of them psychologists and researchers, with previous experience in the subject field, and who had no prior contact with the participants. This study is complementary to a quantitative study whose objective is to analyze the PHCRC, specifically in each of its domains, by assessing people with mental disorders, using the brief questionnaire provided by the WHO Multi-country Survey Study on Health and Health System’s Responsiveness (MCSS) in Spanish [24].

Both participating patients and GPs were recruited from PHC centers in the city of Zaragoza (Spain). Due to the universal nature of the health system in Spain and the absence of other primary health care providers, the data obtained in the study is considered to be representative of the population that met the criteria for inclusion in the study. The Spanish health care system has full coverage of the registered population and is funded by public taxes. The health system patients do not need to pay any sum of money to receive health care or diagnostic tests. This characteristic is common in many European and world health care systems.

Participating patients´ inclusion criteria were outlined as the following: being over 18 years old and having received care from the primary care health professionals (general practitioners or nurses) during the previous 12 months at the time of the study. A subsample of participating patients was required to have suffered from at least one psychiatric illness (generalized anxiety disorder, depression, schizophrenia, addictions, and/or personality disorder), as classified by the ICPC-2 classification framework. They needed to have said illness also registered in their electronic medical records. They had to have received pharmacological and/or psychological treatment, too. The exclusion criteria were the following: not being able to respond to the interviewer, presenting cognitive impairment from any cause (which appears in the patient’s medical history), residing in the area for less than 3 months during the year, and receiving palliative care.

In the sample, 28 patients were used, 17 (46.4%) being women, and 11 (53.6%) men, with an average age of 52.04 years (SD:14.14). Of these 28 patients, 15 suffered from a mental health disorder and 13 did not suffer any. Of the 15 participating subjects with a mental health disorder, 5 were male. This sampling was used in order to analyze differences in both groups of patients in regard to the topic of the study. In the sample, 21 family doctors from different urban health centers, belonging to different population profiles, were also recruited with 1 being a member of a management team. Of the family doctors, 12 were women (57.1%) and 9 were men (41.9%), with an average age of 54.24 (SD:9.48). The average years of experience was 25.67 (SD:10.75).

Participating patients, with and without mental health disorders, were invited to participate in the study according to the order in which they appeared on the participating family doctor’s consultation list. Their sex and age were analyzed as they were included in the study to ensure that there were no significant differences in both groups (*p*-value: 0.448 with regard to sex, p-value: 0.085 with regard to age). The patient’s age (< 40; 41–60;> 60), sex, and mental health pathology were taken into account. The participating family doctors ages (< 40; 41–60;> 60), sex, and years of experience (< 15; 15–30;> 30) were also taken into account with the purpose of obtaining the widest variety of information possible.

All patients, family doctors, and managers initially agreed to be interviewed, and all of them partook in said interviews. Table 2 shows the main characteristics of the 49 participants (28 patients and 21 professionals, of which 20 were GPs and 1 was a member of management from a primary care team and was also a GP). Two focus groups were carried out with the participation of the 20 GPs and an individual interview with the member of the management team. The interviews were conducted from January to July 2019.

**Table 2:** Characteristics of participating patients and health care professionals

Variables	Patients (***n*** = 28)
*Age*	
20–40 years	6 (21.4%)
41–60 years	15 (53.6%)
> 60 years	7 (25%)
*Sex*	
male	11 (39.3%)
female	17 (60.7%)
*Group*	
With mental disorder	15 (53.6%)
Without mental disorder	13 (46.4%)
**Variables**	**Health care professionals** **(*n*** **= 21)**
*Age*	
20–40 years	3 (14.3%)
41–60 years	14 (66.7%)
> 60 years	4 (19%)
*Sex*	
male	9 (41.9%)
female	12 (57.1%)
*Years of experience*	
< 15 years	3 (14.3%)
15–30 years	12 (57.1%)
> 30 years	6 (28.6%)

Health care professional includes GPs and a member from a PC management team

A standardized protocol was designed to guide individual and group interviews. It included the preparation of a topic list to be addressed, with previously tested, open suggestions that could have been of interest. The topic list was based on the guide created by the WHO for qualitative assessment to study patient perception towards their experiences with health services (focus group instrument) [25], and which was translated and back-translated from English to Spanish to ensure the quality of the questions. The patients were asked to detail whether they had ever needed to consult a health care center (and if so, whether it was because of mental health problems). In the case that the patients had not further detailed their experience, they were asked about how they felt in that situation, how long ago that experience happened, how the doctors/nurses treated them, what they thought of the place where they received medical care, and if they could change anything about this experience, apart from their health being better or not, what would they change. These questions were related to positive and negative experiences during their use of PHC services (if the patient reported a negative experience, they were subsequently asked for a positive one and vice versa). The WHO document is intended for focus groups, but in our study, we opted for its use in individual interviews in order to maintain confidentiality, since several of the participants belonged to the same health center and the inclusion criterion of a subsample was to have a diagnosis of mental disorder. In addition, open questions were prepared based on the domains outlined in the Multi-country Survey Study on Health and Health System’s Responsiveness (MCSS) questionnaire [24]. The questions in the MCSS were formulated to be answered on an ordinal scale (how often or how would you rate), while the interviews were formulated with open-ended questions to avoid yes/no answers and referring to health personnel (doctors, nurses, and administrative staff). Table 3 shows the list of topics and questions. The individual interviews with the patients were carried out first, followed by the group interviews with family doctors, and lastly the interview with a member from the management team. Interviews were continued until all possible information was collected.

**Table 3:** Topic list and questions

Topic list	Questions for patients
Before the interview	1. Greetings, words of thanks and introduction of the interviewer and observer. 2. General information about the topic to be discussed and the purpose of the session. 3. Explanation of ethical aspects: confidentiality and informed consent and permission to record. 4. Explanation of the dynamics of the interview (We will ask some questions to find out about your experiences. We are interested in your opinion. Before we continue, do you have any questions, do you have any doubts? Do you agree to participate?)
1. Perception of the response capacity given by the primary care unit.	Could they describe a situation when they went to see a health professional (either a doctor or a nurse) at their health center about a mental health problem? How did they feel in that situation? How long ago did the incident they are describing happen (if they are recounting a negative situation or incident)? How did the doctors/nurses treat them? What do they think about the place where they received care? If they could change anything about this experience, apart from it being better or not, what would they change? If it was a negative experience, ask them to talk about a positive experience and vice versa. What would they expect their health center to be like? Could the health center change anything in the way they are treated?
2. Specific perception of each domain.	After explaining the concept of responsiveness and each domain, ask specifically about their perceptions of the last year, when they have needed attention from their doctor or nursing staff at their health center, and their opinions in the context of each of the domains.
3. Most important domains for patients on responsiveness.	Which of these domains do they consider most important? Why?

* Questions for health care professionals only focus on the second point. Patients with a mental health disorder, who also presented another chronic pathology, were specifically asked in their mental health-related consultations

The objectives of the study were indirectly addressed and questions asked about the topics were answered in an open and progressive way. The interviewers and/or moderators were introduced to the participants as research psychologists and assumed the minimal role of orientating, limiting their interventions to addressing the topics in the script. The environment for data collection was a neutral room in the different health centers in which the patients were registered, without the presence of non-participants in order to ensure the confidentiality of their responses. In-depth interviews lasted between 20 and 60 min and the discussion groups lasted 40–75 min. All sessions were digitally audio-recorded and a verbatim transcription was made in order to obtain the final set of qualitative data for analysis, which was revised by some participants and added to the field notes made during and after the interviews/groups. Participants agreed to participate in the study and signed a consent form. None of the interviews were repeated.

With the intention of assessing the scope of discourse, an inductive thematic content analysis was carried out in order to explore, develop, and define the emergent categories of analysis which derived from the individual interview and group data [26]. This analysis was performed by two researchers independently and was agreed upon between the two where there were discrepancies in the analyses. Subsequently, these emerging categories were recoded based on the theoretical framework developed with consideration to the WHO, HSRC, and previous studies. All analysis was performed iteratively using Maxqda-2007 software in agreement between two researchers, and the interpretations made from the data were discussed with interviewers and participants to obtain their consent [27]. This methodological triangulation was able to increase consistency and rigor by combining multiple techniques and maximizing the breadth and depth of the interpretations carried out.

## Results

Regarding patients’ experiences in their use of Primary Care (PC) services, both negative and positive experiences fell into the categories of dignity, communication, and rapid service. Patients relate dignity and communication very closely in their discourse. Subjects with mental health disorders report more negative experiences regarding dignity, including also recording experiences with management staff. In patients with a mental health disorder, extreme cases of loss of dignity can occur which, although not frequent, tend to be more common in hospital care than in Primary Care. Nevertheless, family doctors recognize that these situations regarding loss of dignity and autonomy can occur with these patients. Although some patients report some negative experiences in relation to dignity and communication, patients without mental health problems generally express that their negative experiences are centered on rapid service. When the participants were asked what they would change, none considered it to be a structural problem. Rather, they considered it to be a problem with certain professionals. Those who have had relevant problems in this respect have solved them by changing professionals. On the other hand, the professionals consider that it is important to take these non-medical aspects into account and to act accordingly during consultations. However, it is often the very organization of the system and the very nature of the profession (dealing with the health and illness of people who each have their own context) that make it complicated to implement non-medical aspects.

In the analysis, no differences were observed in the perception of the PHCRC from the professionals according to their gender. However, in the group of patients, women, regardless of whether or not they suffered from a mental health disorder, reported more negative experiences in terms of communication. As for participants with MD, only one male reported negative experiences regarding clear communication and dignity, while women with MD reported more negative experiences regarding dignity and clear communication.

*"Well, I kept crying and they sent me to hospital because I was too nervous, and once they tied me to... to the stretcher"*

*(Patient, female, 29 years old, with SMD)*

*"We make decisions consulting with the police. Nothing is further from autonomy than to force someone into a hospital or to immobilize them..."*

*(Family doctor, male, >30 years experience)*

*"one thing is your personal behavior and another is how the system allows you to act. I believe that the system has many flaws in terms of autonomy, confidentiality, dignity... I, as a professional, can be very much in favor of this, but sometimes the system does not allow me, or is not inclined to allow me to act in a certain way".*

*(Family doctor, female, >30 years experience)*

### Domain of dignity

When analyzing the questions relating to each of the domains, with respect to dignity, there is a majority perception by patients that they are treated with dignity. However, there are some patients with mental health disorders who specifically comment on some negative experiences with respect to this domain. This fact could be complementary to the perspective of the professionals. They report that if the patient displays aggression or anxiety, has some personality disorder, and/or communicates guilt to the professional, the professional should maintain control of the consultation. However, some circumstances, such as working under pressure, can make the situation more difficult and not satisfactory for either the professional or the patient. Patients also regard this as a two-way street, that is, patients consider that if they address the health professional with respect, the professional must treat them with respect and vice versa. Another aspect that emerged in the discourse of both users and professionals is that the presence of residents and medical students, with whom they do not have close contact and who are often present during exams, etc., is violent to them. However, in this respect doctors say that they usually ask the patient if they agree with the presence of doctors in training. Finally, the difficulty in maintaining privacy during clinical exams also appears in the discourse. Due to small consultation rooms, the positioning of available furniture, or consultation spaces shared with nursing staff, even if screens are used, interruptions can complicate efforts to maintain privacy during exams.

*“For me, it was very good. I also have to say that I, too, am well mannered”.*

*(Patient, female, 70 years old, with SMD)*

*"Sometimes, due to the circumstances surrounding the consultation, and perhaps on a particular day, there may be a feeling of pressure; that everything is going too fast, that the professional does not conduct the consultation well... There may be a certain moment when the doctor is no longer fully in command of their ability to attend in a calm and attentive manner. If at that moment a specific patient who also requires special attention for mental health reasons, for social conditions, or for any other reason particular to their personality, etc., etc., there may be an interaction that may not result in being the most appropriate ... Things happen in a more stagnant and difficult way which may result in the patient or the professional not being satisfied..."*

*(Family doctor, male, >30 years experience)*

*“yes, yes, regardless of the health problem, the treatment of the patients always, generally speaking, but this is also supported by all the satisfaction surveys, is always in principle, very satisfactory”.*

*(Manager, male, 15–30 years experience)*

### Domain of confidentiality

Regarding confidentiality, all patients, regardless of whether they have a mental disorder or not, recognize the importance of confidentiality with no reports having been made about having problems in this regard. According to the professionals, the most challenging aspect is inter-consultation with other professionals. They believe they have established mechanisms to improve confidentiality but there are still aspects to improve. In their discourses, family doctors reported some negative experiences when recording confidential matters regarding the patients (for example, an issue of gender violence), which were being seen to by the health center and in consultation with a specialist intervening in an action already underway at the PC. There are also problems of confidentiality with users who suffer from a mental health disorder who are also seen to by private health care providers since information is not shared. In the groups of professionals, there is no agreement as to whether certain types of pathology with a strong social stigma, for example, severe mental health disorders, HIV, social problems, etc., would have to be reported to all professionals or reserved to only some, with there being opinions for and against the matter.

*“I think so. I don't have any proof to the contrary."*

*(Patient, female, 72 years old, no SMD)*

*"We have so much information and it passes through so many channels, that the truth is that absolute confidentiality is entirely impossible, right? In fact, a detail that just sums up what happens is just how many times a patient tells us ‘hey, I've been sent to god knows who and it says here that I have this health problem and I don't want that to appear on my record’. Patients come to you and they tell you these things. I think it's so difficult. For most people the intention is, supposedly, to maintain confidentiality, but it is exhausting to try to guarantee 100% confidentiality with these systems.”*

*(Family doctor, woman, >30 years experience)*

### Domain of autonomy

With respect to the domain of autonomy, both patients and professionals note patient involvement, when willing, in decisions about their care or treatment, and that their permission should be requested before tests or treatments are performed. However, it also appears that patients often do not want to be involved, regardless of whether they suffer from a mental health disorder or not.

*“There has been a paradigm shift in Medicine, especially in Family Medicine. We have gone from paternalism to a model of autonomy”.*

*(Manager, male, 15–30 years experience)*

*“Everything is explained to them... Sometimes they are not told "do you agree?", but there is an implicit request of... “Do you want me to do this to you?”... or “this should be done”...”*

*(Family doctor, female, 15–30 years experience)*

*“Patients often don't want to get involved... There is a certain delegation of responsibility to the doctor.”*

*(Family doctor, female, >30 years experience)*

### Domain of rapid service

With regard to rapid service, except at specific times of the year, waiting times are short. Usually, there is an appointment on the same day for the family doctor if there is no possibility of continuous care. It does appear repeatedly in all patient discourse that there is a delay with the time of entry to the consultation, but they often attribute this to the fact that the time per consultation is scarce and, therefore, it is normal that there are delays. Professionals emphasize the need for sufficient time in consultations to provide adequate assistance and resolution capacity. With respect to mental health disorders, centers that have a mental health unit integrated note that by having more fluid communication with specialists, patient attention can be faster. There are tools such as minimum waiting time specifications. With regard to waiting for additional tests, which are generally blood tests, rapid service is well perceived.

*“Some countries are establishing quick consultations... They have a system set up where the patient talks to, and is responded to, by the doctor through a computer. I don't think that's the solution, I think there needs to be adequate time, and not quick consultations that don't solve the problem”.*

*(Family doctor, male, >30 years experience)*

### Domain of clear communication

With regard to clear communication, in general, the patients interviewed, at the time of the study, considered that they had good communication and were listened to well by the health professionals. There were patients who reported that when they did not have good communication, they opted to change professionals. Patients also reported negative experiences in terms of communication with administrative staff. On the other hand, the professionals consider that patients are provided with information about their problem in an understandable manner, have close dialogue, and are listened to carefully. However, these professionals also comment that often that there is not enough time to provide this level of service. Professionals also believe that this domain with patients with a severe mental health disorder is more complicated for several reasons. For example, explaining both the prognosis and its implications is more complicated and requires more time, especially for those with severe disorders. Another reason is that the patient may already have a limited capacity of understanding (they come to the consultation without their legal guardian, or in the acute phase of a psychotic episode, etc.).

*"the one at the front desk screeching at me, as if she were the teacher scolding a child. It was practically the same thing!"*

*(Patient, female, 55 years old, with SMD)*

*“The time we give to explain things to the patient comes at the expense of our own health...”*

*(Family doctor, female, 15–30 years experience)*

*“I think the biggest problem is the lack of time we have to talk to patients and families with mental health problems because there are patients which you have for six minutes or ten minutes and you know you need to be there for at least an hour!”*

*(Family doctor, woman, >30 years experience)*

### Domain of choice

With regard to the domain of choice, 25% of the patients interviewed had changed their family doctor at some time and had not had any problems in making this change and choosing another doctor. The main reason why people had changed doctors was because of problems with dignity and communication. The geographical distribution of resources must also be taken into account, as it is different to exercise choice in an urban health center where there are several professionals, than in rural centers in small municipalities where there is only one doctor. Another fact to highlight is that patients are able to change their family doctor depending on the understanding, trust, and doctor-patient relationship they have, but in mental health units located in the health centers, they cannot choose a mental health specialist. There is also no possibility of choice with respect to the administrative staff, and patients have reported negative experiences with this part of the health center staff.

*“In general, in those centers where it is possible, there is no problem”.*

*(Manager, male, 15–30 years experience)*

### Domain of quality of services

With regard to the quality of services, in general, there is the consensus that all is correct and adequate in terms of facilities and their hygienic conditions, however, there also appear to be opinions from patients who feel that cleaning is not correctly done or that it has worsened after the economic crisis. Yet, it has also been mentioned the notion that the responsibility for the care and cleanliness of the facilities also falls back on the patients themselves and there are people who do not take on this responsibility to care for the common facilities. The opinion of the professionals and the health care manager is that it depends on the health centers. There are health centers where there are no complaints about this during the assessment of the facilities in the satisfaction surveys, but there are also others which receive many complaints.

*“There’s room for improvement with this because after going through a period of economic crisis with insufficient funding in Primary Care it’s mmm worsened, or at least put on the backburner with respect to other needs. So, logically there has been some deterioration in the state of the spaces”.*

*(Manager, male, 15-30 years experience).*

*“Before they weren’t any..., it is clear that there have been cuts”.*

*(Patient, female, 51 years old, with SMD).*

*“There is no respect, there are people who don't pay any attention to anything”.*

*(Patient, male, 55 years old, with MD).*

### Relevance of the domains

Regarding the importance of the domains in patients with mental health disorders, the domains, appearing in order of highest to lowest importance, are dignity, rapid service, confidentiality, and clear communication, with 80% accounting for the first two; dignity and communication. For people who do not have a mental health disorder, dignity is also the most important domain (also around 80%), but the domains that follow are confidentiality, autonomy, and rapid service. The latter is not so much of a priority for control participants, while autonomy is. Only 13% of subjects with a mental health disorder place autonomy among the most prominent domains. The domains that are considered less relevant are quality of services and choice. This is also shared by professionals and management. The interrelationship between domains also appears in the discourses with there being mention of a relationship between clear communication, autonomy, dignity, and the latter with confidentiality.

*“Dignity is fundamental and goes hand in hand with confidentiality... Then, there would also be rapid service. Clear communication would come before autonomy because it would be difficult for the patient to choose between different options if I am not able to make clear what they are to them”.*

*(Manager, male, 15–30 years of experience)*

*“Dignity, confidentiality and rapid service”.*

*(Family doctor, female, >30 years of experience)*

*“Clear communication is also the basis for autonomy and dignity. Dignity is also related to confidentiality”.*

*(Family doctor, male, >30 years of experience)*

**Fig. 1:**
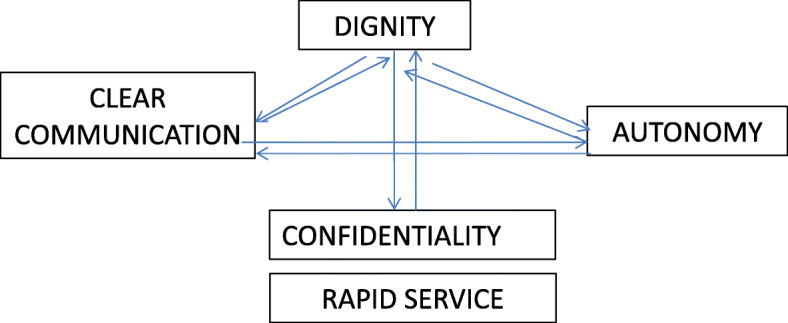
Importance and relationship among domains obtained in the discourses

The domains’ prioritization and their interrelationship is shown in Fig. 1.

## Discussion

The HSRC measures the patients’ experiences regarding non-medical health problems and their legitimate expectations when they come into contact with the health system, evaluating their objective experience with these services, regardless of whether they are satisfied or not with the attention they receive [13, 28]. Mental health care is especially relevant since patients with a mental disorder are especially vulnerable to not being treated as they wish due to the characteristics of their illness and the associated stigma [29, 30]. The qualitative method used in this study is based on the recommendation to use a qualitative research method in order to investigate experiences with non-medical care and individualized disease, and the providers´ experiences in regards to relationships between patients and providers [31].

Regarding the results obtained, dignity, communication, and rapid service are the domains highlighted as important by patients with a mental disorder. The close relationship between dignity and communication was also commented on. These domains were also identified as relevant in the qualitative study that Forouzan carried out in Iran with mentally ill patients [32], as well as in the study by Murante et al. [33]. These findings are also consistent with the fact that people in mental health units are less likely to report that staff treat them with dignity and respect than those in primary and secondary care [34].

Regarding communication, it should be emphasized that women have higher expectations regarding communication [35] and are more likely to prefer a collaborative style of communication with their physicians by taking an active role in the process of their medical care [36]. Research also suggests that both health professionals and patients with a severe mental disorder have difficulty in communicating effectively about symptoms, drug treatments, side effects, and about how to reach a mutual understanding on the topics of diagnosis, prognosis, and treatment [37]. Regarding the training in communication skills, a seminal paper published in the Lancet in 1980 highlighted the potential benefit of teaching communication skills in undergraduate medical programs [38]. Since the time of the study, programs or subjects on communication have been implemented in undergraduate training in health care degrees. However, communication between primary health care personnel and patients with mental health problems is made difficult by the fact that a collaboratively negotiated diagnosis and treatment is needed, taking account of the patient’s preferences, expectations, understanding, and social context. This collaboratively negotiated diagnosis and treatment is difficult to provide with limited time and resources, something for which university education does not prepare [39]. Besides, this difficulty in communication could be due also to the stigma of mental disorders that also exists among healthcare professionals [40].

Regarding the domain of dignity, there is a majority perception by patients that they are treated with dignity, but both patients and health professionals express that treatment must be respectful on both sides. Therefore, authors such as Ziedonis et al. [41] advocate for a supportive and respectful physician-patient alliance as a means of ensuring this dignity.

Regarding confidentiality, the majority of patients have had positive experiences; results which are endorsed in other studies [20, 21, 42, 43]. In general, patients know that confidentiality is the duty of all professionals involved in medical care in the health field [44]. Patients with mental health problems consider this domain to be more relevant compared to patients who do not have mental health problems. This may be related to the stigmatization of mental health [40, 45]. A substantial desire to maintain privacy during consultations also emerged in statements regarding the physical layout of the facility. The physical layout of the facility also emerged in the assessment of dignity at the center as well as in the study by Njeru et al. [42].

Regarding the domain of autonomy, it appears to be a significant domain for all, but specifically for patients without mental health disorders. Patient participation is increasingly recognized as a key component in the redesign of health care processes and is upheld as a means to improve patient safety [46]. However, on many occasions, physicians do not evaluate accurately the patients’ preferences [47]. Furthermore, the autonomy domain is also closely related to clear communication [28] and this communication is more deficient with patients with mental health disorders [37]. This may explain why patients with mental health problems do not place autonomy as a preferred domain.

Our study has important strengths but also has its limitations. Among the strengths, it can be noted that, comparing with patients without mental health problems, a very relevant issue has been addressed for the attention of health service providers in relation to populations suffering from health problems which are highly-prevalent worldwide, such as mental health problems. Another strength is the methodology we employed, since it allowed us to deepen our understanding of patient perception of response capacity. Moreover, it allowed for the number and profile of the participants, collecting of the patient perspectives (service users), health care professionals (service providers), and administrators (managers), and was in line with the principles of the stakeholder theory. This fact allows us to analyze discourse, collecting the opinions from all profiles involved in the health care. However, this study also has limitations such as the very nature of the concept of responsiveness in the health system and in the domains, since the patients are not usually familiar with the notion. Another limitation of this study is that it is necessary to consider its results in the context of the health system in which they occur and according to the sample size. Several studies [33] suggest that HSRC varies substantially between countries due to variations between health systems in each country. On the other hand, although 28 patients and 21 professionals were interviewed, this is a small number to take into account with respect to its external validity. In addition, the aspect of the perception of the PHCRC according to gender and especially in the group of patients with mental health problems should be further investigated, since the results cannot be considered conclusive in this respect and further research would be necessary. It is also necessary to reinforce the perspective given from managers.

In all medical specialties, including Primary Health Care and Mental Health Care, evidence is growing that there is a gap between what is considered optimal care and what is actually provided [19]. Therefore, it is relevant to evaluate and compare the performance of health services and specifically mental health attention, by using a universal indicator, which does not need any adjustment for specific risks, such as the response to the domains found in health services [19]. In addition, it is important to incorporate patients in the generation of knowledge about health systems, and to involve them in health care planning with a view to co-production in health with contextualized, biopsychosocial and salutogenic care models. This participation improves health outcomes and is useful for citizens and professionals to analyze, understand, debate, and decide collectively in order to improve living conditions and environments.

## Conclusions

The fundamental domains for patients, with or without a mental health disorder, are the domains of dignity, communication, and rapid attention, with dignity being closely related to communication. People with mental health problems also highlight the domain of confidentiality as relevant, while patients who do not have a mental health problem prioritize the domain of autonomy. Patients with mental health disorders report a greater number of negative experiences in relation to the domain of dignity. These findings are also endorsed by health care professionals.

## Data Availability

The data that support the findings of this study are available from the corresponding author, (B.O.B)], upon reasonable request.

## Abbreviations

GPs: General Practitioners
MD: Mental disorder
HSRC: Health Systems’ Response Capacity
MCSS: Multi-country Survey Study on Health and Health System’s Responsiveness
PHC: Primary Health Care
PHCRC: Primary Health Care Response Capacity
